# One-way flow over uniformly heated U-shaped bodies driven by thermal edge effects

**DOI:** 10.1038/s41598-022-05534-y

**Published:** 2022-02-04

**Authors:** Satoshi Taguchi, Tetsuro Tsuji

**Affiliations:** 1 Department of Advanced Mathematical Sciences, Graduate School of Informatics, Kyoto University, Kyoto, 606-8501 Japan; 2 Research Project of Fluid Science and Engineering, Advanced Engineering Research Center, Kyoto University, Kyoto, 606-8501 Japan

**Keywords:** Nanoscience and technology, Nanofluidics, NEMS

## Abstract

The thermal edge flow is a gas flow typically induced near a sharp edge (or a tip) of a uniformly heated (or cooled) flat plate. This flow has potential applicability as a nonmechanical pump or flow controller in microelectromechanical systems (MEMS). However, it has a shortcoming: the thermal edge flows from each edge cancel out, resulting in no net flow. In this study, to circumvent this difficulty, the use of a U-shaped body is proposed and is examined numerically. More specifically, a rarefied gas flow over an array of U-shaped bodies, periodically arranged in a straight channel, is investigated using the direct simulation Monte-Carlo (DSMC) method. The U-shaped bodies are kept at a uniform temperature different from that of the channel wall. Two types of U-shaped bodies are considered, namely, a square-U shape and a round-U shape. It is demonstrated that a steady one-way flow is induced in the channel for both types. The mass flow rate is obtained for a wide range of the Knudsen numbers, i.e., the ratio of the molecular mean free path to the characteristic size of the U-shape body. For the square-U type, the direction of the overall mass flow is in the same direction for the entire range of the Knudsen numbers investigated. For the round-U type, the direction of the total mass flux is reversed when the Knudsen number is moderate or larger. This reversal of the mass flow rate is attributed to a kind of thermal edge flow induced over the curved part of the round-U-shaped body, which overwhelms the thermal edge flow induced near the tip. The force acting on each of the bodies is also investigated.

## Introduction

In a gas in which the molecular mean free path is not vanishingly small compared to the system characteristic length, that is, in a rarefied gas, a steady flow is induced thermally in the absence of external forces (e.g., gravity). Examples of thermally induced flow, peculiar to a rarefied gas, include the thermal stress slip flow^1,2^, the nonlinear thermal stress flow^2,3^, and the thermal edge flow^4–6^ in addition to the classical thermal creep flow (or the thermal transpiration)^7–11^.

The thermal creep flow is induced along a nonuniformly heated wall. In contrast, the thermal stress slip flow occurs along a wall when the distance between neighboring isothermal lines (or surfaces) of the gas varies along the wall. The nonlinear thermal stress flow is induced when the distance between neighboring isothermal lines varies along the lines in the interior of the gas, provided the temperature variation is considerable. These properties have been clarified by a systematic asymptotic analysis of the Boltzmann equation with small Knudsen numbers^12,13^. Here, the Knudsen number, denoted by ${{\mathrm {Kn}}}$, is the ratio between the mean free path of the gas molecules and the characteristic length of the system. According to the theory, the magnitude of the flow velocity divided by the thermal speed is of the order of ${{\mathrm {Kn}}}$, ${{\mathrm {Kn}}}^2$, and ${{\mathrm {Kn}}}$, for the thermal creep flow, thermal stress slip flow, and nonlinear thermal stress flow, respectively. Note that when the temperature variation of the gas is small, the nonlinear thermal stress flow is negligibly small compared to the other two flows.

As mentioned, the above classification is based on the asymptotic theory of the Boltzmann equation (in the presence of boundaries). The asymptotic theory assumes that the wall temperature variation and the shape of the boundary are smooth. Therefore, if one of these conditions is violated, another type of flow is possible. The thermal edge flow is of this kind. An abrupt temperature variation near a heated edge (or tip) introduces a strong anisotropy in the momentum transfer to the tip, which results in a flow near the tip by reaction^13^. In this paper, we are concerned with the application of the thermal edge flow with an emphasis on the possibility to induce a one-way flow, which is potentially significant, e.g., for microelectromechanical systems (MEMS)^14,15^.

**Figure 1:**
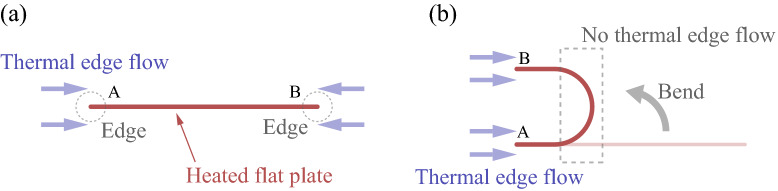
Schematics of the concept. (**a**) A uniformly heated flat plate. A thermal edge flow is induced at each tip (i.e., edge) in the direction depicted in the figure, resulting in no net flow. (**b**) A uniformly heated U-shaped body. The tips A and B induce thermal edge flows in the same direction, leading to a rightward net flow. Note that the curved part is unlikely to induce a thermal edge flow.

The thermal edge flow is, typically, induced near the edges of a uniformly heated (or cooled) flat plate. It is known that the magnitude of the flow velocity is proportional to ${{\mathrm {Kn}}}^{1/2}$ when the Knudsen number is small^5,6,13^. Here, the Knudsen number is taken to be the ratio between the molecular mean free path and the size of the plate. Thus, though localized near the edge, the order of magnitude of the flow velocity is more significant for the thermal edge flow than for the thermal creep flow, which makes it attractive for various applications. Moreover, unlike the thermal creep flow, there is no need to maintain the temperature gradient of the plate, making it easy to use in practice^13,15^. In this regard, a prototypical thermal edge pump has been fabricated in ref.^16^, in which heated and unheated arrays of plates are arranged cleverly so that a net flow is produced. Later, the use of structured channels with ratchet-like topography was proposed with numerical verification, e.g., in refs.^17–19^.

In the original thermal edge flow, a single flat plate is insufficient to induce a net mass flow in a particular direction because forward and backward thermal edge flows from each end cancel out each other (see Fig. 1a). Now, suppose that a flat plate is bent so that it forms a U-shaped body, as shown in Fig. 1b. Then, the two tips are aligned, and the other side of the object is of a round (or curved) shape. In this situation, each tip will induce a thermal edge flow toward the curved side when heated, but the curved part is unlikely to induce a counter flow. Thus, we expect a net flow to occur from the tips toward the curved side, or a one-way flow to occur if the U-shaped bodies are arranged repeatedly in a channel. Motivated by this consideration, in this study, we investigate a rarefied gas flow over periodically arranged U-shaped bodies using the direct simulation Monte-Carlo (DSMC) method. We will demonstrate that a one-way flow is induced over the array of the U-shaped bodies. Further insights into the flow property are also obtained by considering two types of U-shaped bodies, i.e., a square-U type and round-U type.

As shown in this work, each U-shaped body in the array is subject to a force. The situation is similar to the radiometric force exerted on a heated plate^6,20,21^. Thus, if the array is freely movable along the channel, a motion of the array will be induced upon being heated. In this connection, it would be worth mentioning a Crookes radiometer with cup-shaped vanes^7^. The present analysis can be viewed as a model of a cup-shaped-vane radiometer.

The rest of the paper is organized as follows. The formulation of the problem is given in “Formulation,” in which two types of U-shaped bodies are introduced. Subsequently, we present numerical results and related discussions in “Results and Discussion.” First, we consider the case in which a single U-shaped body is confined in a closed vessel and perform a numerical analysis to grasp basic flow properties. Then, we consider a rarefied gas flow over an array of U-shaped bodies and carry out numerical simulations for this case. The mass flow rate and the force acting on the body are then discussed. Finally, we present conclusions in “Concluding Remarks”.

## Formulation

### Problem

**Figure 2:**
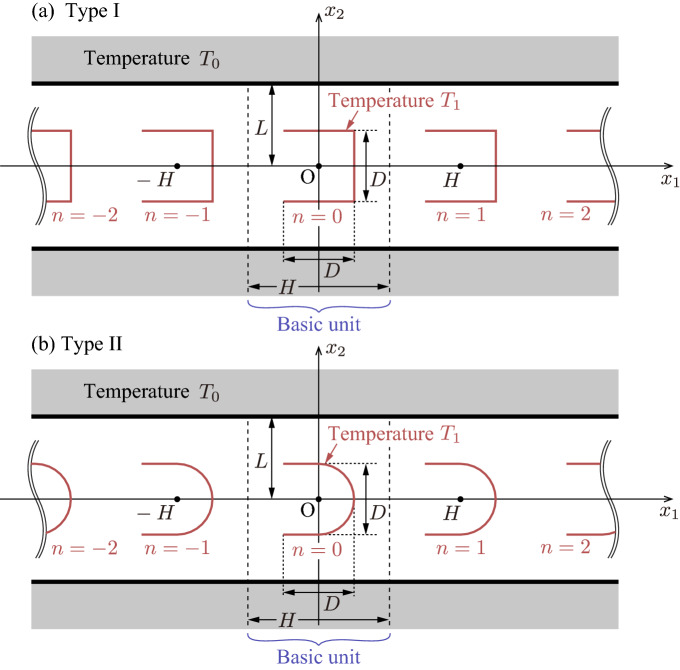
Schematics of the problem: (**a**) type I and (**b**) type II. In panel (**a**) [or (**b**)], infinitely many square-U (or round-U) bodies are arranged periodically along the $x_1$-axis with period *H* between two parallel plates. The U-shaped bodies, located at $nH - \frac{D}{2} \le x_1 \le nH + \frac{D}{2}$ ($n=0$, $\pm 1$, $\pm 2$, $\ldots$), are uniformly heated at a constant temperature $T_1$, while the plates are kept at a uniform temperature $T_0$ ($T_1>T_0$). In both type I and type II, the part $-\frac{H}{2}< x_1 < \frac{H}{2}$ and $-L< x_2 < L$ forms a basic unit.

Let us consider a two-dimensional straight channel between two parallel plates at $x_2= L$ and $x_2 = -L$ kept at a uniform temperature $T_0$ filled with a rarefied gas ($x_i$, $i=1,2,3$, are Cartesian coordinates). The parallel plates are separated by the distance 2*L*. Inside the channel, infinitely many U-shaped bodies are arranged periodically along the $x_1$-axis with period *H*, as shown in Fig. 2. In this paper, we consider two types of (two-dimensional) U-shaped bodies.

- Type I (square-U type): Type I (also called the square-U type) consists of two horizontal line segments with length *D* joined by a vertical line segment (length *D*) (see Fig. 2a).
- Type II (round-U type): Type II (also called the round-U type) consists of two horizontal line segments with length *D*/2 joined by a semicircle with radius *D*/2 (see Fig. 2b).

The U-shaped bodies are kept at a uniform temperature $T_1 (> T_0)$. There is no external force acting on the gas. In this situation, a flow is induced thermally around each body. We investigate the steady behavior of the gas in the channel under the following assumptions:

1. The behavior of the gas is described by the Boltzmann equation for a hard sphere gas.
2. The gas molecules impinging on the plates or the bodies leave the boundary according to the corresponding part of the stationary Maxwellian distribution with temperature and density given by the temperature of the wall and by the impermeability condition across the surface, respectively. In other words, we impose the diffuse reflection boundary conditions^13^ on the plates and on the bodies.

In the present study, we assume that the state of the gas is uniform in the $x_3$ direction (i.e., $\frac{\partial }{\partial x_3} = 0$).

### Basic equations

Let $\rho _{\mathrm {av}}$ be the average density of the gas in the domain $-H/2< x_1 < H/2$ and $-L< x_2 < L$ and let $p_0=\rho _{\mathrm {av}}R T_0$ be the reference pressure, where $R=k_{\mathrm {B}}/m$ is the specific gas constant with $k_{\mathrm {B}}$ the Boltzmann constant and *m* the mass of a gas molecule. Besides the notation introduced in “Problem,” we denote the molecular velocity by $\varvec{\xi } = (\xi _1,\xi _2,\xi _3)$, the velocity distribution function of the gas molecules by $f(x_1,x_2,\varvec{\xi })$, the mass density of the gas by $\rho$, the flow velocity by $v_i$ ($i=1,2,3$), the temperature of the gas by *T*, the pressure of the gas by *p*, and the stress tensor by $p_{ij}$ ($i,j=1,2,3$). The steady Boltzmann equation for a hard sphere gas in the present spatially two-dimensional problem is written as

1a $$\begin{aligned}&\xi _1\frac{\partial f}{\partial x_1} + \xi _2\frac{\partial f}{\partial x_2} = \frac{d_{\mathrm{m}}^2}{2m} \int \left( f^\prime f_*^\prime - f\,f_*\right) |\alpha _j (\xi _{*j}-\xi _j)|\, {\mathrm {d}}\Omega (\varvec{\alpha }) {\mathrm {d}}\xi _{*1}{\mathrm {d}}\xi _{*2}{\mathrm {d}}\xi _{*3}, \end{aligned}$$

1b $$\begin{aligned}&f = f(\xi _i) ,\quad f^\prime = f(\xi _i^\prime ) ,\quad f_*^\prime = f(\xi _{*i}^\prime ), \quad f_*= f(\xi _{*i}), \end{aligned}$$

1c $$\begin{aligned}&\xi _i^\prime = \xi _i + (\xi _{*j}-\xi _j)\alpha _j\alpha _i, \quad \xi _{*i}^\prime = \xi _{*i} - (\xi _{*j}-\xi _j)\alpha _j\alpha _i, \end{aligned}$$

where $d_{\mathrm{m}}$ is the molecular diameter, $\varvec{\alpha } = (\alpha _1,\alpha _2,\alpha _3)$ denotes unit vectors, and ${\mathrm {d}}\Omega (\varvec{\alpha })$ denotes the solid angle element in the direction of $\varvec{\alpha }$; the integral in (1a) is carried out over the whole space for $\varvec{\xi }_* = (\xi _{*1},\xi _{*2},\xi _{*3})$ and all directions for $\varvec{\alpha }$. Note that the functional dependency of *f* on $(x_1,x_2)$ is omitted in (1b).

The diffuse reflection boundary conditions on the two parallel plates are written as

2a $$\begin{aligned}{}&f=\frac{\sigma _{w0}}{(2 \pi R T_0)^{3/2}} \exp \left( -\frac{|\varvec{\xi }|^2}{2 R T_0}\right) , \quad \xi _2\lessgtr 0 \qquad (-\infty< x_1 < \infty ,\;x_2=\pm L), \end{aligned}$$

2b $$\begin{aligned}{}&\sigma _{w0} = {\left\{ \begin{array}{ll} \displaystyle \sqrt{\frac{2\pi }{R T_0}} \int _{\xi _2 > 0} \xi _2 f {\mathrm {d}}\varvec{\xi } &{} (-\infty< x_1< \infty ,\;x_2 = L), \\ \displaystyle - \sqrt{\frac{2\pi }{R T_0}} \int _{\xi _2< 0} \xi _2 f {\mathrm {d}}\varvec{\xi } &{} (-\infty< x_1 < \infty ,\;x_2 = -L), \end{array}\right. } \end{aligned}$$

where ${\mathrm {d}}\varvec{\xi }={\mathrm {d}}\xi _1 \mathrm {d}\xi _2 {\mathrm {d}}\xi _3$. Here and in what follows, the upper and lower signs go together.

The diffuse reflection boundary condition on the U-shaped body in the basic unit $-H/2< x_1 < H/2$ and $-L< x_2 < L$ is described as follows. Let the U-shaped bodies of type I and II be denoted by $D^{\mathrm{I}}$ and $D^{\mathrm{II}}$, respectively, that is,

3a $$\begin{aligned} D^{\mathrm{I}} = & \,\left\{ (x_1,x_2)\, \Big | \, -\dfrac{D}{2}< x_1< \dfrac{D}{2},\quad x_2 = \pm \frac{D}{2} \right\} \nonumber \\&\bigcup \left\{ (x_1,x_2)\, \Big | \, x_1 = \dfrac{D}{2},\quad -\dfrac{D}{2}< x_2 < \dfrac{D}{2} \right\} , \end{aligned}$$

3b $$\begin{aligned} D^\mathrm{II} = &\,\left\{ (x_1,x_2)\, \Big | \, -\dfrac{D}{2}< x_1< 0,\quad x_2 = \pm \frac{D}{2} \right\} \nonumber \\&\bigcup \left\{ (x_1,x_2)\; \Big | \; - \dfrac{D}{2}< x_2 < \dfrac{D}{2},\quad x_1 = \sqrt{\left( \dfrac{D}{2} \right) ^2 - x_2^2} \right\} . \end{aligned}$$

**Figure 3:**
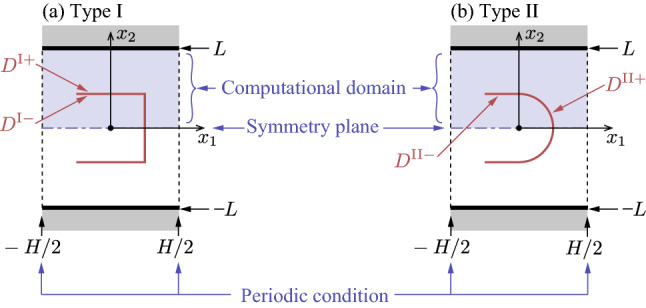
The computational domains in the basic unit for (**a**) type I and (**b**) type II.

We also denote by $D^{J+}$ and $D^{J-}$, $J=\mathrm I$ or II, the convex and concave sides of $D^{J}$, respectively (see Fig. 3). Then, the unit normal vector $n_i^+$ (or $n_i^-$) on $D^{J +}$ (or $D^{J -}$) pointing to the gas are given by

(Type I)

4a $$\begin{aligned} n_i^\pm (x_1,x_2) = {\left\{ \begin{array}{ll} (0,\pm 1,0), &{} -\dfrac{D}{2}< x_1< \dfrac{D}{2},\quad x_2 = \dfrac{D}{2} \pm\, 0, \\ (0,{\mp } 1,0), &{} -\dfrac{D}{2}< x_1< \dfrac{D}{2}, \quad x_2 = -\dfrac{D}{2} {\mp }\, 0, \\ (\pm 1,0,0), &{} x_1 = \dfrac{D}{2} \pm\, 0,\quad - \dfrac{D}{2}< x_2 < \dfrac{D}{2}, \end{array} \right. } \end{aligned}$$

(Type II)

4b $$\begin{aligned} n_i^\pm (x_1,x_2) = {\left\{ \begin{array}{ll} (0,\pm 1,0), &{} -\dfrac{D}{2}< x_1< 0,\quad x_2 = \dfrac{D}{2} \pm\, 0, \\ (0,{\mp } 1,0), &{} -\dfrac{D}{2}< x_1< 0,\quad x_2 = -\dfrac{D}{2} {\mp } \,0, \\ \dfrac{\pm (x_1,x_2,0)}{\sqrt{x_1^2 + x_2^2}}, &{} - \dfrac{D}{2}< x_2 < \dfrac{D}{2},\quad x_1 = \sqrt{\left( \dfrac{D}{2} \right) ^2 - x_2^2} \pm \,0, \end{array}\right. } \end{aligned}$$

where the upper (or lower) sign corresponds to $D^{J+}$ (or $D^{J-}$). In the sequel, $n_i$ represents either $n_i^+$ or $n_i^-$. With this preparation, the boundary conditions on the U-shaped bodies in the basic unit are summarized as

5a $$\begin{aligned}{}&f(x_1,x_2,\xi _i) = \frac{\sigma _{w1}^\pm (x_1,x_2)}{(2 \pi R T_1)^{3/2}} \exp \left( -\frac{|\varvec{\xi }|^2}{2 R T_1}\right) , \quad \xi _i n_i^\pm >0, \ (x_1,x_2) \in D^{J\pm }, \end{aligned}$$

5b $$\begin{aligned}{}&\sigma _{w1}^\pm (x_1,x_2) = -\sqrt{\frac{2\pi }{R T_1}}\int _{\xi _i n_i^\pm < 0} \xi _i n_i^\pm f(x_1,x_2,\xi _i) \mathrm {d}\varvec{\xi }. \end{aligned}$$

Since the U-shaped bodies are periodically arranged with the uniform interval *H* in the current setting, we look for a periodic solution with period *H*, that is,

6 $$\begin{aligned} f(x_1,x_2,\xi _i) = f(x_1+H,x_2,\xi _i). \end{aligned}$$

In other words, we restrict ourselves to the basic unit $-H/2< x_1 < H/2$ and $-L< x_2 < L$ and impose the periodic boundary conditions at $x_1 = \pm H/2$ (see Fig. 3). Moreover, since $x_2 = 0$ is the symmetry plane, we shall analyze the problem in the upper half domain by imposing the following specular reflection at $x_2=0$:

7 $$\begin{aligned} f(x_1,0,\xi _1,\xi _2,\xi _3) = f(x_1,0,\xi _1,-\xi _2,\xi _3), \quad \xi _2>0. \end{aligned}$$

The solution in the lower half domain is obtained by the mirror image of that in the upper half domain, that is, $f(x_1,x_2,\xi _1,\xi _2,\xi _3) = f(x_1,-x_2,\xi _1,-\xi _2,\xi _3)$ for $-L< x_2 < 0$.

Once the velocity distribution function *f* is obtained by solving the boundary-value problem (1), (2), and (5), the macroscopic quantities are given as the moments of *f*:

8 $$\begin{aligned} \begin{aligned}{}&\rho = \int f\mathrm {d}\varvec{\xi }, \quad v_i = \frac{1}{\rho }\int \xi _i f\mathrm {d}\varvec{\xi }, \quad RT = \frac{1}{3\rho }\int (\xi _i-v_i)^2 f\mathrm {d}\varvec{\xi }, \\&p = \rho R T, \quad p_{ij}=\int (\xi _i- v_i)(\xi _j- v_j) f\mathrm {d}\varvec{\xi }, \end{aligned} \end{aligned}$$

where the range of integration is the whole space of $\varvec{\xi }$. To evaluate the pumping performance, the mass flow rate per unit length in the $x_3$ direction is introduced as $M_f$, i.e.,

9 $$\begin{aligned} M_f = \int _{-L}^{L} \rho v_1 \mathrm {d}x_2. \end{aligned}$$

Furthermore, we introduce the force $F_{i}$ acting on each of the U-shaped body per unit length in the $x_3$ direction:

10 $$\begin{aligned} F_{i} = - \int _{\Sigma } p_{ij} n_j \mathrm {d}S, \end{aligned}$$

where $\mathrm {d}S$ is the surface element and the integration is carried out on the surface $\Sigma =D^{\mathrm {I}+} \cup D^{\mathrm {I}-}$ (or $\Sigma =D^{\mathrm {II}+} \cup D^{\mathrm {II}-}$) for type I (or type II); see the sentence below Eq. (3). Note that the geometrical symmetry leads to $F_{2}=F_{3}=0$ for both type I and II.

Finally, we list the physical parameters that characterize the problem. They are summarized as

11 $$\begin{aligned} \frac{T_1}{T_0}, \quad {\mathrm {Kn}}=\frac{\ell _0}{D} \quad \left( \ell _0 = \frac{1}{\sqrt{2} \pi d_\mathrm{m}^2(\rho _\mathrm {av}/m)}\right) , \quad \frac{H}{D}, \quad \frac{L}{D}, \end{aligned}$$

where we have introduced the Knudsen number ${\mathrm {Kn}}$ with $\ell _0$ being the mean free path of the gas molecules at the reference equilibrium state at rest. The DSMC simulation is carried out for various sets of these parameters. Further details of the simulation are given in “Method.”

## Results and discussion

In the present paper, the geometric parameters are fixed to $H/D=2$ and $L/D=1$. There should be some optimal parameter set (*L*/*D*, *H*/*D*) for given $T_1/T_0$ and ${\mathrm {Kn}}$ that maximizes $M_f$. Nonetheless, in this paper, we focus on the effects of ${\mathrm {Kn}}$ and $T_1/T_0$, and, in particular, the impact of the difference between the square and round-U types.

### Case of a single body

Before presenting the main results, we first consider the case in which a single U-shaped body is confined in a two-dimensional rectangular vessel filled with a rarefied gas and investigate the flow induced thermally in the vessel as a preliminary. To be more specific, we consider a U-shaped body (type I or II) placed in a vessel enclosed by planes $x_1 = \pm H/2$ and $x_2 = \pm L$ kept at a uniform temperature $T_0$. The U-shaped body is located in the region $-D/2 \le x_1 \le D/2$ and $-D/2 \le x_2 \le D/2$ and is kept at a uniform temperature $T_1 (>T_0)$. We investigate the steady behavior of the gas induced in the vessel under the same assumptions as expressed in “Formulation,” that is, the molecules make diffuse reflections on the sidewalls of the vessel as well as on the upper and lower walls. This situation is appropriate for investigating the qualitative behavior of the gas flow induced by the uniformly heated U-shaped body because there is no interaction between neighboring bodies inherent in the original problem. The parameters are the same as those shown in Eq. (11), and we will show the results for the case $H/D=2$, $L/D=1$, and $T_1/T_0=2$.

**Figure 4:**
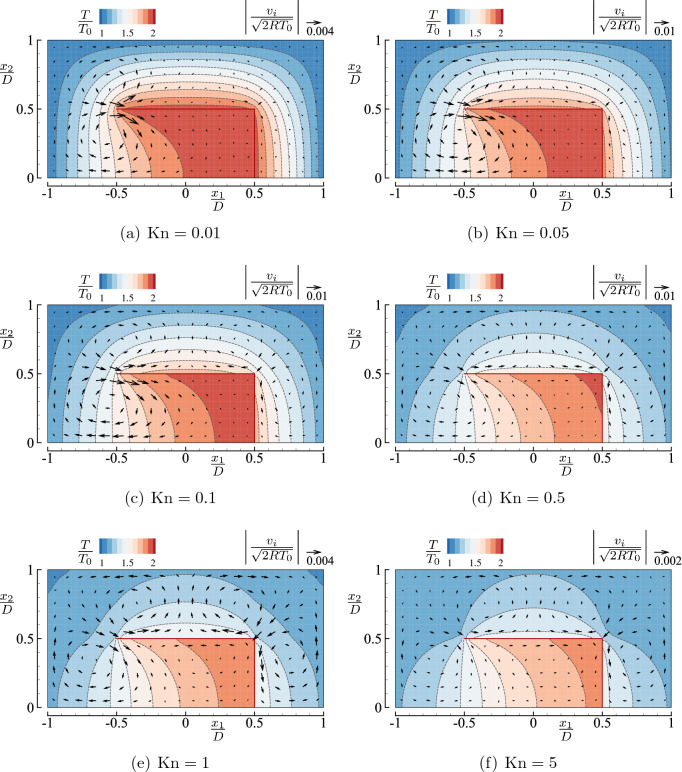
Flow velocity vector $(v_1,v_2)$ and contour map *T* in the case of a single square-U body in a closed vessel ($T_1/T_0=2$, $H/D=2$, and $L/D=1$) for typical Knudsen numbers ${\mathrm {Kn}}=$ (**a**) 0.01, (**b**) 0.05, (**c**) 0.1, (**d**) 0.5, (**e**) 1, and (**f**) 5. Note that the magnitude of the reference vector is different in panels (**a**), (**e**) and (**f**).

Figure 4 shows the flow velocity vector $(v_1,v_2)$ and the contour curves of the temperature *T* for the square-U body case, in which (a) ${\mathrm {Kn}}=0.01$, (b) 0.05, (c) 0.1, (d) 0.5, (e) 1, and (f) 5. In panels (a), (e), and (f), the flow speed is much smaller than those in panels (b)–(d). Therefore, the magnitude of the reference vector is smaller in panels (a), (e), and (f) to magnify the velocity vector for better visibility. It is seen from the figure that a significant flow is induced near the tip $(x_1/D,x_2/D)=({-0.5},{0.5})$ in the rightward direction when ${\mathrm {Kn}}\lesssim 0.5$. The magnitude of this flow becomes relatively large when ${\mathrm {Kn}}\approx 0.1$ and decreases as ${\mathrm {Kn}}$ is further increased. There is another relatively strong flow near the right corner $(x_1/D,x_2/D)=({0.5},{0.5})$ along the top and side plates when ${\mathrm {Kn}}\gtrsim 0.1$. The corner flow is attributed to the same mechanism as the thermal edge flow and its flow direction over the top side is opposite to the edge flow near the tip $(x_1/D,x_2/D)=({-0.5},{0.5})$. This leftward corner flow is weaker than the thermal edge flow near the tip when ${\mathrm {Kn}}$ is small, but it competes well against the rightward edge flow when ${\mathrm {Kn}}\gtrsim 0.5$. Consequently, we observe four circulating flow patterns when $0.1 \lesssim {\mathrm {Kn}}\lesssim 1$, whose centers are located near $(x_1/D,x_2/D)\approx ({-0.5},{0.25})$ (clockwise), $(x_1/D,x_2/D)\approx ({-0.5},{0.75})$ (counter clockwise), $(x_1/D,x_2/D)\approx ({0.5},{0.75})$ (clockwise), and $(x_1/D,x_2/D)\approx ({0.75},{0.25})$ (counter clockwise), see Fig. 4d,e. At ${\mathrm {Kn}}=5$, the flow velocity is considerably reduced with the roll structure remaining unchanged. The magnitude of the flow velocity is most significant for (c) ${\mathrm {Kn}}=0.1$ and (d) ${\mathrm {Kn}}=0.5$ and tend to vanish with a further increase of ${\mathrm {Kn}}$ (see the last two paragraphs of this subsection for more quantitative discussions).

The contour curves of the temperature *T* are concentrated near the tip when ${\mathrm {Kn}}$ is small, indicating a steep temperature variation there, as pointed out in refs.^4,6^. As ${\mathrm {Kn}}$ increases, the deviation of the gas temperature from that of the body becomes large, and it is more prominent outside the body than inside. The gas is hotter inside the U shape than outside because the molecules inside have more chances to hit the heated wall and to encounter faster molecules. For high ${\mathrm {Kn}}$ cases (e.g., ${\mathrm {Kn}}=5$), the contour curves are bent near the tip and the corner. The mechanism of such bending of isolines is discussed in the case of a free molecular gas in ref.^22^.

**Figure 5:**
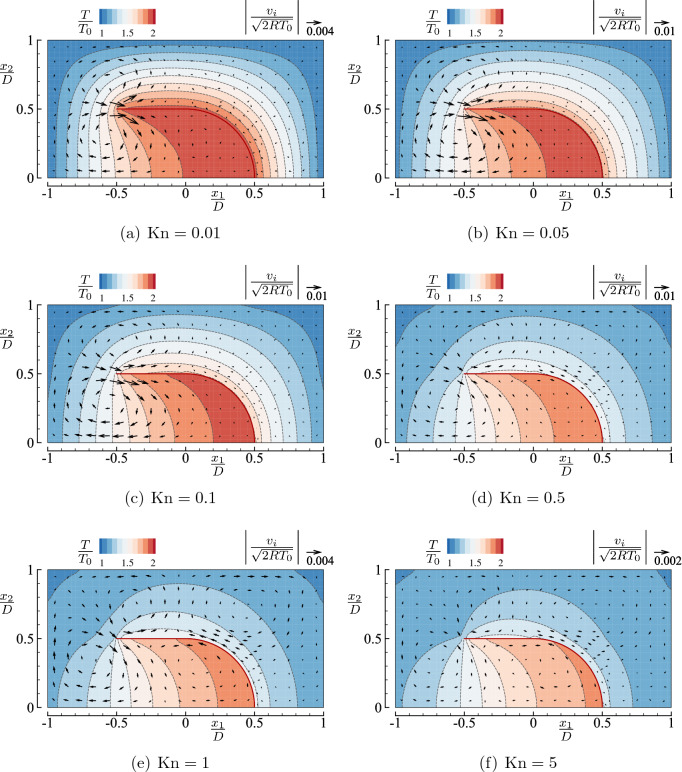
Flow velocity vector $(v_1,v_2)$ and contour map *T* in the case of a single round-U body in a closed vessel ($T_1/T_0=2$, $H/D=2$, and $L/D=1$) for typical Knudsen numbers ${\mathrm {Kn}}=$ (**a**) 0.01, (**b**) 0.05, (**c**) 0.1, (**d**) 0.5, (**e**) 1, and (**f**) 5. See the caption of Fig. 4.

Now, to compare the flow field between the cases of square and round-U bodies, we show in Fig. 5 the corresponding figure for the round-U body. First, we notice that the overall flow structures are similar for the square and round-U types in the left half region, i.e., $x_1<0$. A difference in the flow field is observed around the outer face of the curved part, i.e., $x_1^2+x_2^2\gtrsim (D/2)^2$ with $x_1>0$. When ${\mathrm {Kn}}\le 0.1$, we hardly see the flow in this right half region for the round-U shape. With the increase of ${\mathrm {Kn}}$ $(\ge 0.5)$, a flow in the negative $x_1$ direction becomes noticeable along the outer face of the curved part. This flow is caused by the geometry of the round-U shape (it may be called a blunt edge effect), as discussed later. As we will see later in “Mass flow rate,” the flow in the negative $x_1$ direction overwhelms the flow near the tip and results in a negative pumping. The temperature isolines around the round-U type exhibit a similar bending in the left half domain, as seen in Fig. 4, and a milder bending in the right half domain for high ${\mathrm {Kn}}$ $(=5)$ compared with the square-U type [cf. Figs. 4f and 5f].

**Figure 6:**
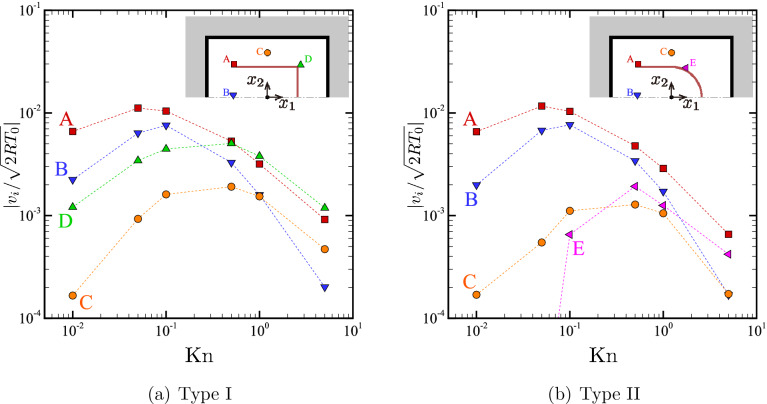
Magnitude of the local flow velocity $|v_i|=(v_1^2+v_2^2)^{1/2}$ versus ${\mathrm {Kn}}$ at a given location in the gas in the closed domain case; (**a**) Type I, (**b**) type II. The locations are as follows. A: $(x_1/D,x_2/D)=(-0.525,0.525)$; B: $(-0.525,0.025)$; C: (0, 0.75); D: (0.525, 0.525); E: (0.25, 0.45). Note that, for the point E in panel (**b**), the values of $|v_i|/\sqrt{2RT_0}$ are less than $10^{-4}$ for ${\mathrm {Kn}}=0.01$ and 0.05, which are of the order of statistical error and therefore not considered to be meaningful.

To support the descriptions given above, Fig. 6 presents the magnitude of the local flow velocity as a function of ${\mathrm {Kn}}$ at four locations in the gas for the types I and II. More precisely, Fig. 6a shows, for the type I body, $|v_i|=(v_1^2+v_2^2)^{1/2}$ versus ${\mathrm {Kn}}$ at the following four locations: (A) $(x_1/D,x_2/D)=(-0.525,0.525)$, (B) $(-0.525,0.025)$, (C) (0, 0.75), and (D) (0.525, 0.525). Similarly, Fig. 6b shows the same quantity for the type II body at the locations (A), (B), (C), and (E) $(x_1/D,x_2/D)=(0.25,0.45)$. At every location, the flow speed increases with ${\mathrm {Kn}}$ when ${\mathrm {Kn}}$ is small, attains its maximum, and decreases with a further increase of ${\mathrm {Kn}}$. The flow speed is the fastest near the tip when the Knudsen number is small, irrespective of the type of U-shape (see point A).

Incidentally, the flow vanishes when ${\mathrm {Kn}}\rightarrow \infty$ in both square and round-U cases, as proved by a general theory for the free molecular flow (see ref.^13^, Chap. 2).

### Flows over a periodic array of U-shaped bodies

In this subsection, we examine the behavior of the macroscopic quantities of the main problem described in “Formulation.” The values of the physical parameters $$({\mathrm {Kn}}$$ and $$T_1/T_0)$$ are the same as those in “Case of a single body”.

**Figure 7:**
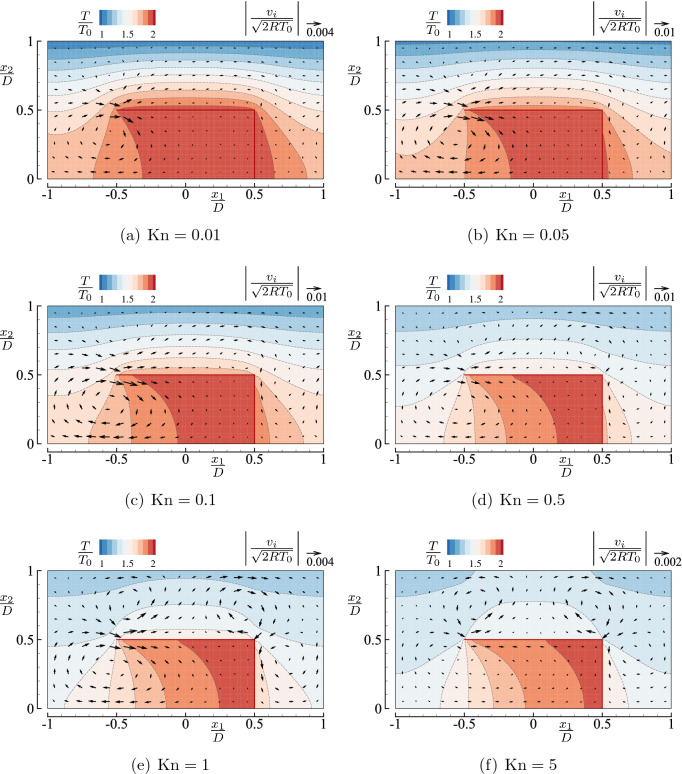
Flow velocity vector $(v_1,v_2)$ and contour map *T* in the case of an array of square-U bodies ($T_1/T_0=2$, $H/D=2$, and $L/D=1$) for typical Knudsen numbers ${\mathrm {Kn}}=$ (**a**) 0.01, (**b**) 0.05, (**c**) 0.1, (**d**) 0.5, (**e**) 1, and (**f**) 5. See the caption of Fig. 4.

First, we show the flow velocity and the temperature field in Fig. 7 for the square-U body array. For relatively small Knudsen numbers ${\mathrm {Kn}}\le 0.1$ (panels (a–c)), we see the onset of a thermal edge flow at $(x_1/D,x_2/D)\approx ({-0.5},{0.5})$ as in the case of a closed vessel (see Fig. 4). We also observe a weaker corner flow near $(x_1/D,x_2/D)=({0.5},{0.5})$. This leftward corner flow is overwhelmed by the rightward thermal edge flow in the region $0< x_1/D < 0.5$ and $0.5< x_2/D <1$. Since the mass flow rate $M_f$ across $x_1={\text {const}}$ should be independent of $x_1$, this means that a net mass flow in the positive $x_1$ direction occurs in the channel. Note that $\int _0^{D/2} \rho v_1 \mathrm {d}x_2 = 0$ at any cross section $x_1 = {\text {const}}$ inside the U-shaped body (i.e., $-0.5< x_1/D < 0.5$ and $0 \le x_2/D<0.5$). The magnitude of the flow tends to decrease with the decrease of ${\mathrm {Kn}}$, as seen from panels (c)$\rightarrow$(b)$\rightarrow$(a). On the contrary, for relatively large Knudsen numbers ${\mathrm {Kn}}\ge 0.5$ (panels (d–f)), the flow patterns near the corner are similar between Figs. 4 and 7; there are two rolls near the corner. In particular, the appearance of two rolls near the tip and the right corner in Fig. 7d–f suggests that the pumping effect, if exists, is weak for these ${\mathrm {Kn}}$ $(\ge 0.5)$. It is also seen that the magnitude of the flow decreases as ${\mathrm {Kn}}$ becomes large (see, panels (d)$\rightarrow$(e)$\rightarrow$(f)).

**Figure 8:**
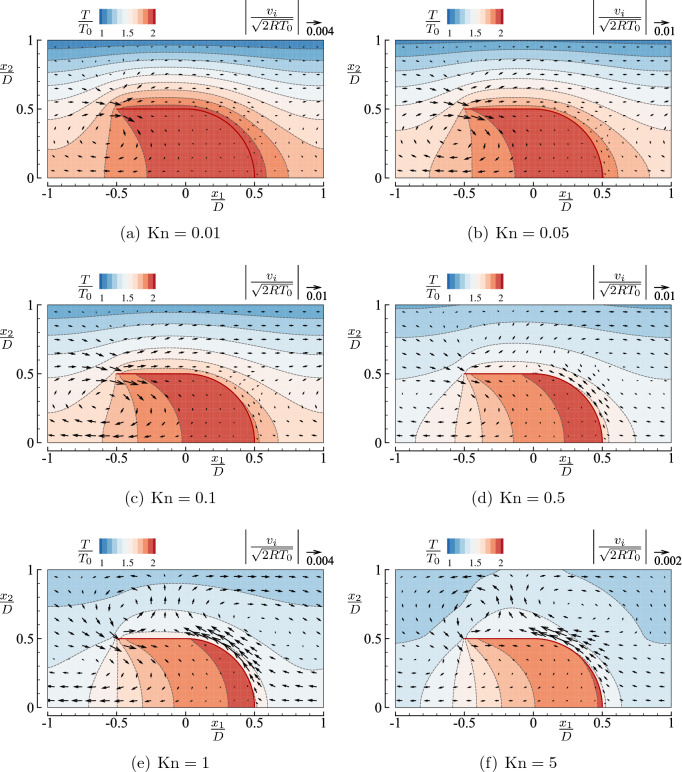
Flow velocity vector $(v_1,v_2)$ and contour map *T* in the case of an array of round-U bodies ($T_1/T_0=2$, $H/D=2$, and $L/D=1$) for typical Knudsen numbers ${\mathrm {Kn}}=$ (**a**) 0.01, (**b**) 0.05, (**c**) 0.1, (**d**) 0.5, (**e**) 1, and (**f**) 5. See the caption of Fig. 4.

The temperature field exhibits the same feature as in the case of a single body presented in Fig. 4. However, since the heated bodies repeatedly appear in the current periodic case, the overall temperature inside the channel is higher.

Now, let us move on to the results for the array of round-U bodies, which are shown in Fig. 8. In the region $0< x_1/D < 0.5$ and $0.5< x_2/D < 1$, we observe a net mass flow in the positive $x_1$ direction for relatively small ${\mathrm {Kn}}\le 0.1$, as in the case of the array of square-U bodies. However, the case of round-U bodies leads to more $x_1$-aligned flow patterns, and the magnitude of the flow in Fig. 8 is more significant than in Fig. 7. For relatively larger ${\mathrm {Kn}}$ $(\ge 0.5)$, the flow along the curved part, directed in the negative $x_1$ direction, is apparent. We have also observed such a flow in the closed case (see Fig. 5). If we look at the flow velocity across a particular plane perpendicular to the channel, e.g., $x_1=H/2$ $(=D)$, we notice that the mass flow rate across the plane seems to be reduced due to this backward flow. The magnitude of the backflow is the largest around ${\mathrm {Kn}}\approx 1$ and decreases when ${\mathrm {Kn}}$ is further increased.

We notice that the temperature is lower inside the round-U shape than inside the square-U shape when ${\mathrm {Kn}}$ is large (see Figs. 7f and 8f). This difference can be understood in the following way. The molecules leaving the channel wall tend to have a slower speed compared with those leaving the U-shaped body. Suppose that there is few inter-molecular collisions. Then, these slow molecules have more chances to reach the point near the closed end inside the U shape in the case of the round-U body than in the case of the square-U body. On the other hand, there is no noticeable difference in the temperature value when ${\mathrm {Kn}}$ is small because the inter-molecular collisions result in more enhanced temperature diffusion.

### Mass flow rate

**Figure 9:**
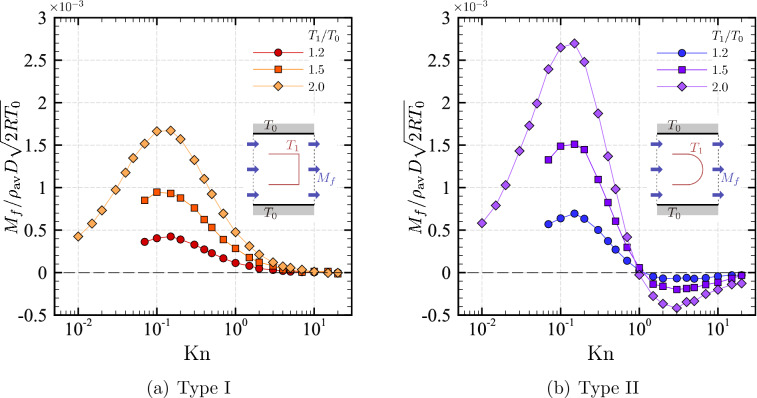
Mass flow rate $M_f$ in the $x_1$ direction per unit length in $x_3$ as a function of the Knudsen number ${\mathrm {Kn}}$ in the cases of $T_1/T_0=1.2$, 1.5, and 2 ($H/D=2$, $L/D=1$). (**a**) Square-U type, (**b**) round-U type. The symbols represent the numerical results, which are joined by the line segments.

We now show the results for the mass flow rate as a measure of pumping performance. We show in Fig. 9 the mass flow rate $M_f$ in the $x_1$ direction per unit length in $x_3$ for various ${\mathrm {Kn}}$ and $T_1/T_0$ (${\mathrm {Kn}}=0.1$, 0.15, ..., 20; $T_1/T_0=1.2$, 1.5, and 2). The results for even smaller Knudsen numbers, ${\mathrm {Kn}}=0.01$, 0.015, ..., 0.07, are also included for the case of $T_1/T_0=2$.

First, let us look at the mass flow rate in the case of type I body, i.e., the square-U shape. When $T_1/T_0=2$, the mass flow rate increases (or decreases) with ${\mathrm {Kn}}$ when the Knudsen number is small (or large). There is a Knudsen number (${\mathrm {Kn}}\approx 0.1$) at which $M_f$ reaches its maximum for given $T_1/T_0$. In this problem, in which the flow is induced by the thermal edge effects, the numerical analysis using the DSMC method becomes increasingly severer as $T_1/T_0$ becomes small, particularly for small ${\mathrm {Kn}}$. (Note that the appreciable flow is more and more localized near the edge as ${\mathrm {Kn}}$ is decreased.) For this reason, the number of numerical results for small ${\mathrm {Kn}}$ is limited in the cases of $T_1/T_0=1.5$ and 1.2. However, we expect the same tendency to occur for smaller values of $T_1/T_0$. Note that $M_f$ takes positive values for all ${\mathrm {Kn}}$ (except possibly noise effects). In other words, the mass flow rate is always in the positive $x_1$ direction in the case of a square-U type.

In the case of type II body, i.e., a round-U shape, the mass flow rate is also increasing in ${\mathrm {Kn}}$ when ${\mathrm {Kn}}$ is small. This trend is observed in the range ${\mathrm {Kn}}\lesssim 0.1$, as in type I body. Note that the values of $M_f$ are considerably larger than those for type I body. We can understand the reason from a difference in the flow pattern observed in Fig. 7 (type I) and Fig. 8 (type II). That is, a leftward (weaker) thermal edge flow induced near the corner partly cancels the rightward thermal edge flow induced near the tip in the case of type I, while there is no such a (backward) thermal edge flow in the case of type II body, when ${\mathrm {Kn}}$ is small.

When ${\mathrm {Kn}}$ is large, the behavior of $M_f$ for type II is qualitatively different from that for type I. In the case of type II, $M_f$ decreases first, becomes negative, and then increases again to zero as ${\mathrm {Kn}}$ increases. In other words, a backward one-way flow occurs when ${\mathrm {Kn}}$ is large for type II. This negative $M_f$ is caused by a thermal “blunt” edge flow induced along the round face as seen in Fig. 8d–f. More precisely, as ${\mathrm {Kn}}$ becomes large, the round face roughly acts as a (blunt) edge in the scale of the mean free path and induces a leftward flow along the heated surface. Moreover, the numerical result shows that this leftward flow dominates the rightward flow. We note that a similar blunt edge flow is also observed around an oval shape body^23^. We also mention that switching between positive and negative mass flow rates for large ${\mathrm {Kn}}$ has also been reported in the case of a structured channel^19^. In general, the behavior of $M_f$ for large ${\mathrm {Kn}}$ is affected by the geometrical configuration of the boundary, and the flow feature is highly problem-dependent.

To summarize, Table 1 shows the maximum value of $M_f$ for given $T_1/T_0$ along with the value of ${\mathrm {Kn}}$ at which the maximum occurs in our computation ($H/D=2$, $L/D=1$).

**Table 1:** Maximum values of $M_f$ with respect to Knudsen number ${\mathrm {Kn}}$ for various $T_1/T_0$ in the case of $H/D=2$ and $L/D=1$.

Type	$T_1/T_0$	${\mathrm {Kn}}$	Maximum of $M_f/\rho _\mathrm {av}D \sqrt{2 R T_0}$
Type I (Square-U)	1.2	0.15	$0.42\times 10^{-3}$
	1.5	$0.1$	$0.95\times 10^{-3}$
	2.0	0.15	$1.67\times 10^{-3}$
Type II (Round-U)	1.2	0.15	$0.69\times 10^{-3}$
	1.5	0.15	$1.51\times 10^{-3}$
	2.0	0.15	$2.70\times 10^{-3}$

### Forces acting on U-shaped bodies

**Figure 10:**
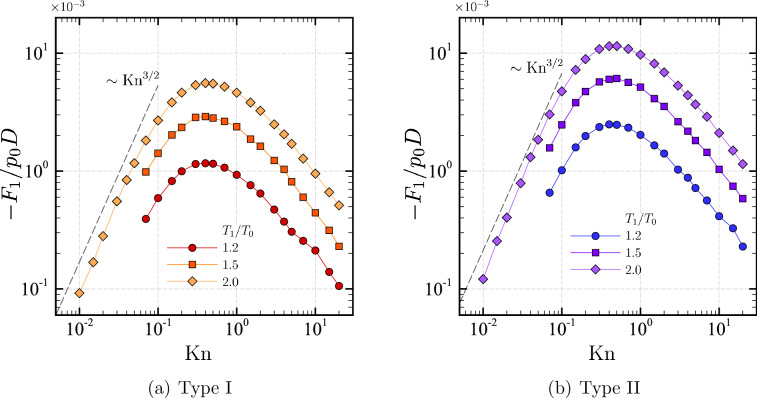
Semi-logarithmic plot of the force $(F_1,0,0)$ acting on each of the U-shape bodies as a function of the Knudsen number ${\mathrm {Kn}}$ for $T_1/T_0=1.2$, 1.5, and 2 in the case of $H/D=2$ and $L/D=1$ ($T_1/T_0=1.2$, 1.5, and 2). (**a**) Type I (square-U shape) and (**b**) Type II (round-U shape). See the caption of Fig. 9.

Finally, we discuss the force acting on the U-shaped body. Figure 10 summarizes the (normalized) force in the $x_1$ direction (per unit length in $x_3$) acting on (a) a square-U type body (type I) and (b) a round-U type body (type II), i.e., $F_1$ in Eq. (10), for various ${\mathrm {Kn}}$ and $T_1/T_0$. The values of ${\mathrm {Kn}}$ and $T_1/T_0$ are the same as those in Fig. 9.

First, we observe that the force is in the direction of negative $x_1$ for both square and round-U types, and its magnitude increases with $T_1/T_0$. Concerning the dependency on ${\mathrm {Kn}}$, the magnitude of the force increases with ${\mathrm {Kn}}$ when ${\mathrm {Kn}}$ is small, attains its maximum at ${\mathrm {Kn}}\approx 0.5$, and then decreases with a further increase of ${\mathrm {Kn}}$. Table 2 shows the maximum value of $-F_1$ obtained in our numerical computations for various $T_1/T_0$, along with the value of ${\mathrm {Kn}}$ at which the maximum is attained. The maximum force acting on the round-U-type body is nearly twice larger than that of the square-U-type body. Indeed, it is found that a more significant force acts on the round-U type for all the Knudsen numbers investigated, as seen from Fig. 10. For small ${\mathrm {Kn}}$, the force likely decreases in proportion to ${\mathrm {Kn}}^{3/2}$ with the decrease of ${\mathrm {Kn}}$ for both types I and II.

Let us focus our attention on the case of small ${\mathrm {Kn}}$. The momentum conservation implies that the thermal edge flow near the tip $(x_1/D,x_2/D)=({-0.5},{0.5})$ in the positive $x_1$ direction pulls the tips (and the body) in the negative $x_1$ direction. Indeed, as we see below, the distribution of the (local) tangential force on the body is leftward (i.e., the negative $x_1$ direction) on the whole horizontal part of the type II body (i.e., round-U type) and almost the entire horizontal part of the type I body (i.e., square-U type). Here, “almost” means that the local force is positive near the right corner located at $(x_1/D,x_2/D)=({0.5},{0.5})$ when ${\mathrm {Kn}}$ is small (${\mathrm {Kn}}\lesssim 0.1$). Near the right corner, another (weaker) thermal edge flow occurs in the negative $x_1$ direction. This counter-flow gives a contribution that partly cancels the negative force in the case of a type I shape.

As shown in the previous subsection, the direction of mass flow rate is reversed when ${\mathrm {Kn}}$ becomes large in type II. However, we do not observe such an inversion of the force acting on the array of round-U-shaped bodies (the total force is always negative, irrespective of ${\mathrm {Kn}}$).

**Figure 11:**
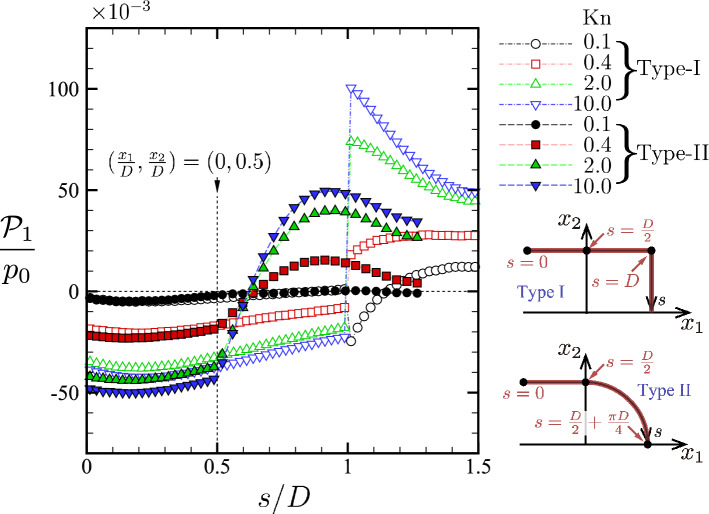
Profile of $\mathcal {P}_1$ (Eq. (13)) along each of the U-shape bodies as a function of the length *s* measured from the edge $(x_1/D,x_2/D)=({-0.5},{-0.5})$ for ${\mathrm {Kn}}=0.1$, 0.4, 2, and 10 in the case of $T_1/T_0=2$, $L/D=1$, and $H/D=2$. The symbols represent the numerical results, which are joined by the line segments. The empty (or filled) symbols indicate the results of type I (or type II). The inset shows the definition of the coordinate *s*.

To provide a further insight into the force acting on the U-shaped body, we look at the distribution of the local stress $p_{1j}n_j$ along the body. For this purpose, we introduce a new coordinate (arc length) along the surface of the body, denoted by *s*, as follows (see also the inset in Fig. 11):

12a $$\begin{aligned}{}&{\text {(Type I)}} \nonumber \\&s = s(x_1,x_2) = {\left\{ \begin{array}{ll} x_1 + \dfrac{D}{2}, &{} -\dfrac{D}{2} \le x_1 \le \dfrac{D}{2}, \quad x_2 = \dfrac{D}{2},\\ D + \left( \dfrac{D}{2} - x_2\right) , &{}\quad x_1 = \dfrac{D}{2}, \quad 0 \le x_2 \le \dfrac{D}{2}, \end{array}\right. } \end{aligned}$$

12b $$\begin{aligned}{}&{\text {(Type II)}} \nonumber \\&s = s(x_1,x_2) = {\left\{ \begin{array}{ll} x_1 + \dfrac{D}{2}, &{} -\dfrac{D}{2} \le x_1 \le 0, \quad x_2 = \dfrac{D}{2},\\ \dfrac{D}{2} + \dfrac{D}{2} \left[ \dfrac{\pi }{2}- \arctan \left( \dfrac{x_2}{x_1}\right) \right] , &{} \quad 0 \le x_1 \le \dfrac{D}{2}, \quad x_2 = \sqrt{\left( \dfrac{D}{2}\right) ^2 - x_1^2}. \end{array}\right. } \end{aligned}$$

Then, the sum of the local stress over two faces $D^{J+}$ and $D^{J-}$ $(J=\mathrm {I},\mathrm {II})$ exerted by the gas, which we denote by $\mathcal {P}_1$, is defined as a function of *s*:

13 $$\begin{aligned} \mathcal {P}_1(s) = - p_{1j} n_j^+|_{(x_1,x_2)\in D^{J+}}- p_{1j} n_j^-|_{(x_1,x_2) \in D^{J-}}. \end{aligned}$$

Note that the integration of $\mathcal {P}_1(s)$ over the entire *s* ($0 \le s/D \le 3/2$ for type I and $0 \le s/D \le (1 + \pi /2)/2$ for type II) leads to half the force $F_1$.

We show in Fig. 11 the distribution of $\mathcal {P}_1(s)$ for types I and II for ${\mathrm {Kn}}=0.1$, 0.4, 2, and 10 in the case of $T_1/T_0=2$. It is seen that the negative component is dominant for the part $0 \le s/D \le 0.5$ for both types of I and II and for all ${\mathrm {Kn}}$, contributing to a leftward force. In particular, the open symbols (type I) and closed symbols (type II) almost overlap when ${\mathrm {Kn}}$ is small (cf. ${\mathrm {Kn}}=0.1$) in the same range, indicating that the local flow structures near the tips are the same for both types I and II in the near continuum regime: a thermal edge flow prevails there for both cases. A difference between type I and type II becomes apparent in the region $s/D>0.5$. For type II, $\mathcal {P}_1$ increases with *s* and changes the sign from negative to positive at some value of *s*. If we magnify the figure (not shown here), we see that the coordinate *s* at which $\mathcal {P}_1$ changes the sign is larger for smaller ${\mathrm {Kn}}$. On the other hand, for type I, $\mathcal {P}_1$ is negative up to $s/D = 1_-$ for ${\mathrm {Kn}}=0.4$, 2, and 10, and suddenly changes to positive values at $s/D = 1_+$ and remains positive for $s/D > 1$. In the case of ${\mathrm {Kn}}=0.1$, $\mathcal {P}_1$ becomes slightly positive in the region $0.9 \lesssim s/D < 1$, jumps to a negative value at $s/D=1_+$, and then goes up to positive values as *s* increases. The decrease of $\mathcal {P}_1$ at $s/D=1_+$ when ${\mathrm {Kn}}=0.1$ is attributed to the thermal stress enhanced by the presence of a sharp corner. We will come back to this point below. The area of the positive part in Fig. 11 is larger for type I than for type II, and this excess positive contribution explains smaller $|F_1|$ for type I.

**Table 2:** Maximum values of $-F_1$ with respect to the Knudsen number ${\mathrm {Kn}}$ for various $T_1/T_0$ in the case of $H/D=2$ and $L/D=1$.

Type	$T_1/T_0$	${\mathrm {Kn}}$	Maximum of $-F_1/(p_0 D)$
Type I (Square-U)	1.2	0.4	$1.16\times 10^{-3}$
	1.5	0.4	$2.90\times 10^{-3}$
	2.0	0.4	$5.58\times 10^{-3}$
Type II (Round-U)	1.2	0.4	$2.49\times 10^{-3}$
	1.5	0.5	$6.10\times 10^{-3}$
	2.0	0.5	$11.47\times 10^{-3}$

Considering that the body’s surface area is smaller for the round-U type than for the square-U type, such an enhancement of the force is helpful in applications for microflows, for which a design for further miniaturization is essential.

**Figure 12:**
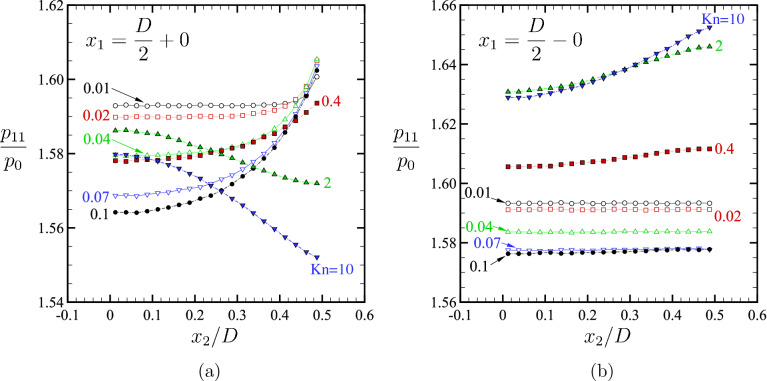
Normal stress $p_{11}$ along the vertical part of the type I shape, i.e., $x_1=D/2\pm 0$ and $0 \le x_2 < D/2$, for various ${\mathrm {Kn}}$ in the case of $T_1/T_0=2$ ($H/D=2$, $L/D=1$). (**a**,**b**) Profiles of $p_{11}$ along (**a**) $x_1=D/2+0$ and (**b**) $x_1=D/2-0$ for various ${\mathrm {Kn}}$. Filled symbols are used for ${\mathrm {Kn}}\ge 0.1$ and empty symbols for ${\mathrm {Kn}}< 0.1$.

Finally, we discuss the cause of the negative jump of $\mathcal {P}_1$ at the right corner observed in the type I body when ${\mathrm {Kn}}$ is small. Figure 12 shows the profiles of the normal stress $p_{11}$ along the outer and inner sides of the vertical segment of the type I body (i.e., $x_1=D/2\pm 0$ and $0 \le x_2 < D/2$) for various ${\mathrm {Kn}}$ ($T_1/T_0=2$). It is seen that, with the decrease of ${\mathrm {Kn}}$, a peak is formed near the corner $x_2=D/2$ on the outer side of the segment [see Fig. 12a]. On the other hand, we observe no formation of such a peak on the inner side; the profile becomes more flattened as ${\mathrm {Kn}}$ decreases [see Fig. 12b]. Therefore, the negative jump in $\mathcal {P}_1$ near $s/D=1$ (see Fig. 11, open symbols $\circ$) is caused by the peak on the outer side near the corner.

Note that a peak-like distribution for $p_{11}$ is also observed for ${\mathrm {Kn}}=0.4$ in Fig. 12a. However, the values of $p_{11}$ are much higher on the concave side than on the convex side of the vertical plate (see Fig. 12b). Therefore, the distribution of $\mathcal {P}_1$ shows no negative jump at $s/D=1$ in Fig. 11 for ${\mathrm {Kn}}=0.4$.

**Figure 13:**
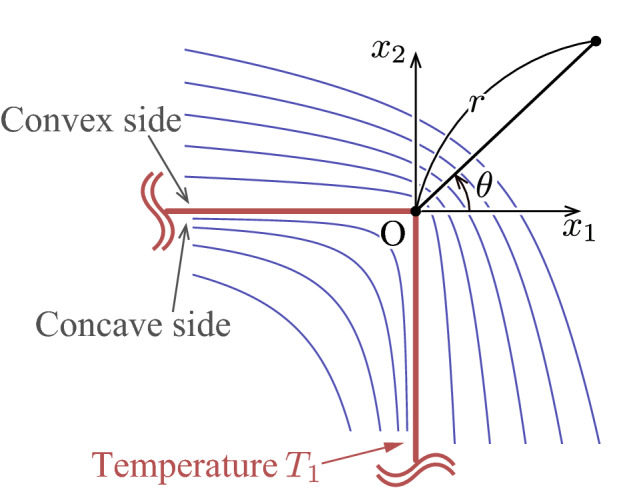
An infinite expanse of a gas separated by a sharp-cornered two dimensional body with a uniform temperature $T_1$. The temperature isolines around the body are schematically shown.

Here, we try to explain the origin of the peak observed in Fig. 12a using a crude estimate of the thermal stress near the corner, following the discussion of ref.^6^. Let us consider an infinite expanse of a gas separated by a corner with a right angle formed by two semi-infinite lines, as depicted in Fig. 13. Suppose that the temperature of the gas is described by a steady heat conduction equation (with a constant thermal conductivity for simplicity). Then, the temperature of the gas around the corner is given for the convex side by

14 $$\begin{aligned}{}&T = T_1 \left[ 1 - a^+\, r^{2/3} \sin \left( \dfrac{2}{3} \theta + \dfrac{\pi }{3}\right) \right] , \quad -\dfrac{\pi }{2}< \theta < \pi , \end{aligned}$$

and for the concave side by

15 $$\begin{aligned}{}&T = T_1 [1 - a^-\, r^2 \sin (2\theta )], \quad \pi< \theta < \dfrac{3}{2} \pi , \end{aligned}$$

where $(r,\theta )$ are polar coordinates with the origin on the corner, $T_1$ is the uniform temperature of the body, and $a^+$ and $a^-$ are constants ($a^+$ and $a^-$ are positive when the body is heated). Now we consider a plane $x_1 ={ \text {const}}$ in the vicinity of the corner and estimate the normal stress $\tau _{11}$ on the plane, which is roughly given by^6^

16 $$\begin{aligned} \tau _{11} \sim T(x_1+ \ell ,x_2) + T(x_1 - \ell ,x_2) \sim 2T|_{(x_1,x_2)} + \frac{\partial ^2 T}{\partial x_1^2}\Big |_{(x_1,x_2)} \ell ^2. \end{aligned}$$

Here, $\ell$ is the mean free path of the gas molecules. Thus, the normal component of the stress tensor is modulated by the term $(\partial ^2 T/\partial x_1^2) \ell ^2$ when a temperature inhomogeneity is present in the gas. This contribution, known as the thermal stress^13^, is usually a small correction when $\ell$ is small. However, it can be large around a sharp edge, where the temperature field varies abruptly, as shown in ref.^6^. In the present case with a sharp corner, a similar enhancement may be possible. In fact, if we take three points $(x_1,x_2) = (0,0)$, $(\ell ,0)$, and $(2\ell ,0)$ in the vicinity of the corner, and estimate $\partial ^2 T/\partial x_1^2$ by the central finite difference formula, we have, using (14),

17 $$\begin{aligned} \frac{\partial ^2 T}{\partial x_1^2} \ell ^2&\sim \frac{T(0,0)- 2 T(\ell ,0) + T(2\ell ,0)}{\ell ^2} \ell ^2 = \frac{\sqrt{3}(2-2^{2/3})}{2} a^+ \ell ^{2/3}. \end{aligned}$$

Therefore, the thermal stress is proportional to $\ell ^{2/3}$, which is more substantial than $\ell ^2$ when $\ell$ is small. Note that a similar estimate applied to the inner side results in no enhancement of the thermal stress (due to the uniform temperature along the horizontal surface), which is also consistent with the behavior of the normal stress presented in Fig. 12b.

**Figure 14:**
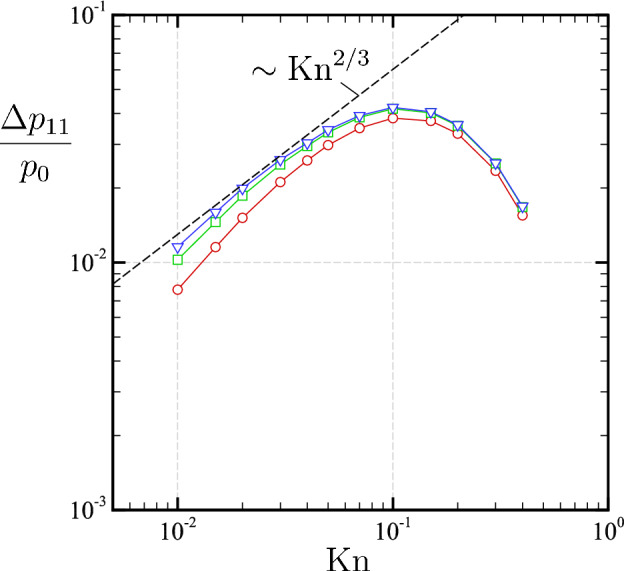
Double-log plot of $\Delta p_{11}\equiv \sup _{0< x_2 < D/2} (p_{11}(x_1=\tfrac{D}{ 2}+0,x_2)-p_{11}(x_1=\tfrac{D}{2}+0,0))$ versus ${\mathrm {Kn}}$. The value of $p_{11}(x_1=\tfrac{D}{2}+0,\frac{1}{2}D)$ is approximated by the node nearest to the corner (red circle), the linear extrapolation using two points near the corner (green square), and the 2nd-order extrapolation using 3 points near the corner (blue lower triangle). Extrapolation is based on the Lagrange formula.

To support the above crude estimate, Fig. 14 shows a double-log plot of

$$\Delta p_{11} \equiv \sup _{0< x_2 < D/2} (p_{11}(x_1=\tfrac{D}{ 2}+0,x_2)-p_{11}(x_1=\tfrac{D}{2}+0,0))$$

as a measure of the peak on the outer side of the vertical segment, as a function of ${\mathrm {Kn}}$ for type I ($T_1/T_0=2$). Since the DSMC method is a cell-based method, it is rather difficult to evaluate the value of $p_{11}$ at the corner $(x_1,x_2)=(\frac{D}{2}+0,\frac{D}{2})$ precisely. Therefore, we extrapolate $p_{11}$ using the values on the nearest cells. As we can see from the figure, the peak decreases in proportion to ${\mathrm {Kn}}^{2/3}$ as ${\mathrm {Kn}}$ is reduced, which is consistent with the above estimate (the thermal stress $\sim \ell ^{2/3}$). In this way, the peak of the normal stress, and thus the sudden change in the local force at the corner, are explained by the enhancement of the thermal stress near the sharp corner when ${\mathrm {Kn}}$ is small.

## Concluding remarks

In this paper, we have considered a steady flow of a rarefied gas over an infinite array of uniformly heated U-shaped bodies. This configuration is motivated by the question of whether one can extract a net flow effectively using a thermal edge flow only by devising the geometry of a body. We addressed this issue numerically using the DSMC method, a standard numerical technique for solving the Boltzmann equation. Two types of U-shaped bodies have been considered and compared to obtain further insights into the flow properties around the U-shape body.

Our computations using the DSMC method show that a one-way flow is induced in the channel for both types. Locally, the flow direction near the tips is such that the tips are facing the induced flow (i.e., the thermal edge flow). The direction of the overall mass flow rate is in the same direction as the thermal edge flow for type I for the entire range of ${\mathrm {Kn}}$ investigated. In type II shape, on the other hand, the mass flow rate is reversed when the Knudsen number becomes large (${\mathrm {Kn}}\gtrsim 1$). A kind of thermal edge flow (or a blunt edge flow) induced along the outer face of the curved part causes this reversal of the mass flow rate, by dominating the rightward thermal edge flow. Thus, there is a threshold of the Knudsen number above which the mass flow rate is reversed for type II. The mass flow rate is generally more significant for type II than type I for the same Knudsen number when they are in the same direction (Fig. 9).

With regard to the force acting on each of the bodies, the force direction is such that it tends to push the body with convex side trailing, irrespective of the value of ${\mathrm {Kn}}$. The force is, in general, larger for the round-U shape than for the square-U shape for the same parameter set (Fig. 10). For type I body, the normal stress along the vertical side of the body exhibits a negative peak near the corner, which is explained by the thermal stress near the sharp corner.

## Method

The simulation is carried out based on a non-dimensional version of the Boltzmann equation (1), in which all the quantities are scaled by suitable reference values. For instance, we use *D* and $\sqrt{2 R T_0}$ as the reference length and speed, respectively.

The DSMC method solves the time-dependent Boltzmann equation and needs an initial condition at time $t=0$. We use the reference equilibrium distribution

18 $$\begin{aligned} f = \frac{\rho _\mathrm {av}}{(2 \pi R T_0)^{3/2}} \exp \left( -\frac{\xi _i^2}{2 R T_0}\right) , \quad t =0, \end{aligned}$$

as the initial condition. The computational domain $\mathcal {V}^J$

19 $$\begin{aligned} \mathcal {V}^J = \left\{ (x_1,x_2) \;\Big |\; -\frac{H}{2}< x_1< \frac{H}{2},\ 0< x_2 < L \right\} \setminus D_{*}^J, \quad J=\mathrm{I, II}, \end{aligned}$$

where $D_{*}^J$ is the set obtained from $D^J$ by restricting the range of $x_2$ to $x_2>0$, is divided into square cells with the area $\Delta x^2$, where $\Delta x/D = 2.5\times 10^{-2}$, i.e., the number of cells is 3200 for the square-U type. In the case of the round-U type, we need additional non-rectangular cells along the curved part. To be more precise, if a square cell has intersections with $D_{*}^{II}$ at two points, the cell is divided into two portions, where the curve of $D_{*}^{II}$ is approximated by a segment that connects these two intersections. These two portions are either (i) two trapezoids or (ii) a triangle and a pentagon, depending on the position of the intersections. The collision process and the computations of moments are carried out using these new cells with areas smaller than $\Delta x^2$. On the other hand, the process of the free transport of the particles is done using the curve of $D_{*}^{II}$. The difference between the curve and the segment is so small that it does not affect the discussion of the paper. For ${\mathrm {Kn}}\le 0.05$, a smaller size of the basic cell with the length $\Delta x/D = 1.25\times 10^{-2}$ is used.

At the initial time, *N* test particles are distributed in each cell, where $N=10^2$ is used. Time step of the simulation is $\Delta t/t_0 = 10^{-3}$ (or $5\times 10^{-4}$) for $\Delta x/D = 2.5\times 10^{-2}$ (or $1.25\times 10^{-2}$), where the reference time $t_0$ is defined as $t_0=D/\sqrt{2 R T_0}$. We judge steady state at $t/t_0=2\times 10^2$ and start sampling at every time step. The number of samples is at least $2\times 10^7$ for all the cases and increased up to $4\times 10^8$ for the cases with small $T_1/T_0$. Note that the macroscopic quantities obtained by the DSMC method are cell-based quantities, that is, any function of $(x_1,x_2)$ (e.g., $\rho$) represents the value in a cell with its center at $(x_1,x_2)$.

To compute the mass flow rate $M_f$ and the force $F_1$, we use the following procedures instead of the direct integration of (9) and (10). In a steady state, the impulse acting on a body over a period $\Delta \bar{t}$ is written as $F_1\Delta \bar{t}$. This should be equal to the summation of the impulse given by a gas molecule that hit the body during the same interval $\Delta \bar{t}$. Since the impulse is computed from the momentum change between before and after the impact, we have

20 $$\begin{aligned} F_1 \Delta \bar{t} = m_*\sum _j (\xi _1^{(j)}-\xi _1^{\prime (j)}), \end{aligned}$$

where $m_*$ is a mass of a test particle, $\xi _1^{(j)}$ and $\xi _1^{\prime (j)}$ are the $x_1$ component of the velocity of the *j*-th particle before and after the impact, respectively, and the summation in Eq. (20) runs over the index *j* for the molecules that hit the body during the interval $\Delta \bar{t}$. The mass flow rate $M_f$ is obtained by counting the number of test particle that goes across a plane $x_1=H/2$ during the interval $\Delta \bar{t}$. To be more specific, let $n_+^{(j)}$ and $n_-^{(j)}$ be the number of translocation from $x_1<H/2$ to $x_1>H/2$ and $x_1>H/2$ to $x_1<H/2$, respectively, of the *j*-th particle during the interval $\Delta \bar{t}$. Then, $M_f$ is given by

21 $$\begin{aligned} M_f\Delta \bar{t} = m_*\sum _{j} (n_+^{(j)}-n_-^{(j)}), \end{aligned}$$

where *j* runs over whole particles intersecting the plane $x_1=H/2$.

## Data Availability

The datasets generated during the current study are available from the corresponding author on reasonable request.
